# Primary Cutaneous Anaplastic Large Cell Lymphoma of the Nasal Dorsum and Nasal Tip

**DOI:** 10.7759/cureus.23811

**Published:** 2022-04-04

**Authors:** Maria C Michali, Alkistis T Tsikou, Charalampos E Tsafaras, Polyniki I Kastanioudaki, Ioannis D Komnos

**Affiliations:** 1 Department of Otorhinolaryngology, Head and Neck Surgery, University Hospital of Ioannina, Ioannina, GRC; 2 Department of Otorhinolaryngology, Head and Neck Surgery, Universith Hospital of Ioannina, Ioannina, GRC

**Keywords:** nasal tip and dorsum, primary cutaneus lymphoma, nasal skin lesion, anaplastic large cell lymphoma, granulomatous-necrotic skin lesion

## Abstract

Primary cutaneous anaplastic large-cell lymphoma (C-ALCL) is described as a non-Hodgkin lymphoma that affects only the skin, with no evidence of an extracutaneous disease during the first six months following the diagnosis. We present an unusual case of a 53-year-old man with developed fever and a rapidly increasing ulcerated skin lesion located on the nasal dorsum and nasal tip. The nasal endoscopy revealed a reddish polypoid lesion over the anterior edge of the superior turbinate which had extended through the roof of the nasal cavity in the adjacent area of the nasal septum.

## Introduction

The anaplastic large cell lymphoma (ALCL) is a neoplasm of activated lymphocytes [1,2]. The primary cutaneous anaplastic large-cell lymphoma (PC-ALCL) is designated as a malignant lymphoma constituted of large cells with an anaplastic, pleomorphic cytomorphology. In addition, an expression of the cluster of differentiation (CD) 30 antigen is observed in more than 75% of tumor cells and there is no expression of anaplastic lymphoma kinase (ALK) in most cases [3,4]. The CD30+ T-cell lymphoproliferative disorders also include lymphomatoid papulosis (LyP) and borderline CD30+ lesions [5]. The CD30 antigens may also be expressed in other lymphoproliferative degenerations like large cell transformations of mucosis fungoides (MF), Hodgkin’s disease, cutaneous NK/T-cell lymphoma, and systemic ALCL. Mucosis fungoides is the most typical form representing 50% of all cutaneous non-Hodgkin lymphomas [5,6].

The PC-ALCL is a rare lymphoma that appears on the skin and the diagnosis should be made when systemic localization has been excluded by adequate staging procedures, including a bone marrow biopsy, according to the criteria of the WHO-EORTC (World Health Organization-European Organization for Research and treatment of Cancer) [3]. The differential diagnosis, treatment, and progression of the disease may conceal perils and pitfalls. PC-ALCL is a disease with an extremely good prediction. Skin lesions can regress spontaneously or occasionally they can relapse. In rare cases, other malignancies may be developed, such as Hodgkin's or non-Hodgkin lymphoma or MF. Thus, a multidisciplinary approach by dermatologists, radiation oncologists, medical oncologists, and hematologists is mandatory and should be preferred [7].

Regarding PC-ALCL, there is a tendency for affecting the male gender (1,5 to 2:1) and can concern people of all ages, with a higher incidence in adults 45 to 60 years old [1,4]. Frequently it is demonstrated as one to multiple nodules greater than 2 cm in diameter, mainly localized. Moreover, erythema and ulceration are very usual findings in this disorder [8].

PC-ALCL can be treated with radiotherapy or, in cases of a small solitary tumor, by surgical removal. Chemotherapy should be reserved for multicentric or extracutaneous types of disease (systemic ALCL). The chemotherapy regimen, that is most commonly used, is CHOP (cyclophosphamide, doxorubicin, vincristine, and prednisone) [3].

## Case presentation

A 53-year-old man with a medical history of arterial hypertension and dyslipidemia appeared at the emergency ward of our hospital with an extensive granulomatous-necrotic skin lesion of the nasal skin with an expansive tendency. The patient was a smoker (one packet/day) and he didn’t report any relevant trauma in the region of the lesion. Four months earlier, an erythematous papule had appeared on the nasal tip’s skin which had developed to an ulcerated lesion. Ιn the last three weeks he had fever (up to 38.5° C every afternoon) and concomitant night sweats. He visited an adjacent hospital where he was examined by a dermatologist, an internist, and an otorhinolaryngologist who proposed systematic medical treatment with cefuroxime for ten days and methylprednisolone for five days. The patient followed the suggested treatment without any clinical improvement.

**Figure 1:**
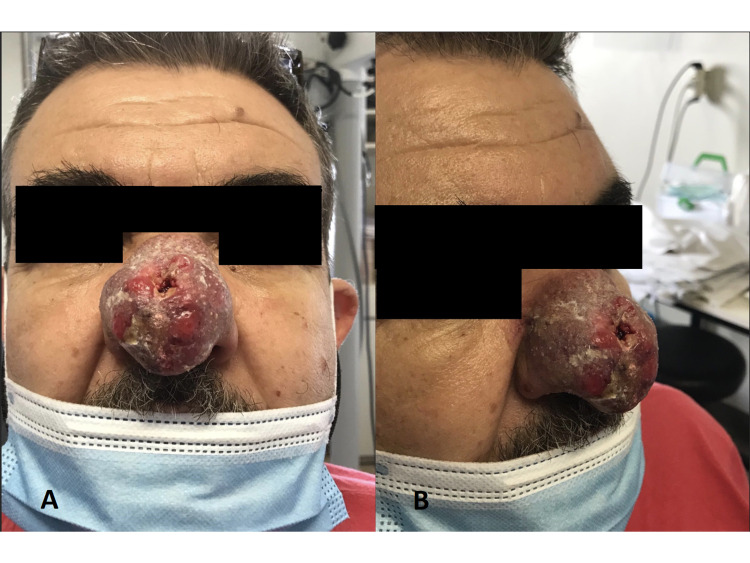
A/B: Ulcerated lesion, covered by necrotic tissue and rounded by erythematous borders.

In parallel, the patient reported no history of cancer, immunosuppressive condition or diabetes mellitus. On physical examination an ulcerated region with raised rubicund borders was observed, involving the nasal tip, the nasal dorsum, the columella and the alars, covered by a necrotic crust. This degenerative tissue was soft in consistency and with no tendency to bleed (Figure 1A, 1B). Additionally, the anterior rhinoscopy and endoscopy of the nose revealed a red lesion on the roof of the nasal cavities and on the adjacent nasal septum’s mucosa. The contigious mucosa over the middle turbinate and over the septum was thick and hemorrhagic.

Laboratory tests for bacteria and virus antibodies including Ebstein-Barr Virus (EBV), Clostridium defficile, Βorrelia , Brucella, Chlamydia, Cytomegalovirus (CMV), Leismania Donovani, Leptospira, Mycoplasma , Toxoplasma, Treponema pallidum, Varicella, Human Immunodeficiency Virus (HIV), Herpes Simplex Virus (HSV), were all negative. The antineutrophil cytoplasmic antibodies (c-ANCA, p-ANCA), antinuclear antibodies (ANA) were negative as well. Furthermore, blood tests were performed. The C-reactive protein (CRP) was 32 (normal value <5) whilst the white blood cells (WBC) was 4.580/μl. The Mantoux test that was conducted, was positive (a significant reaction: 23mm after 72 hours), potentially due to a tuberculosis infection.

**Figure 2:**
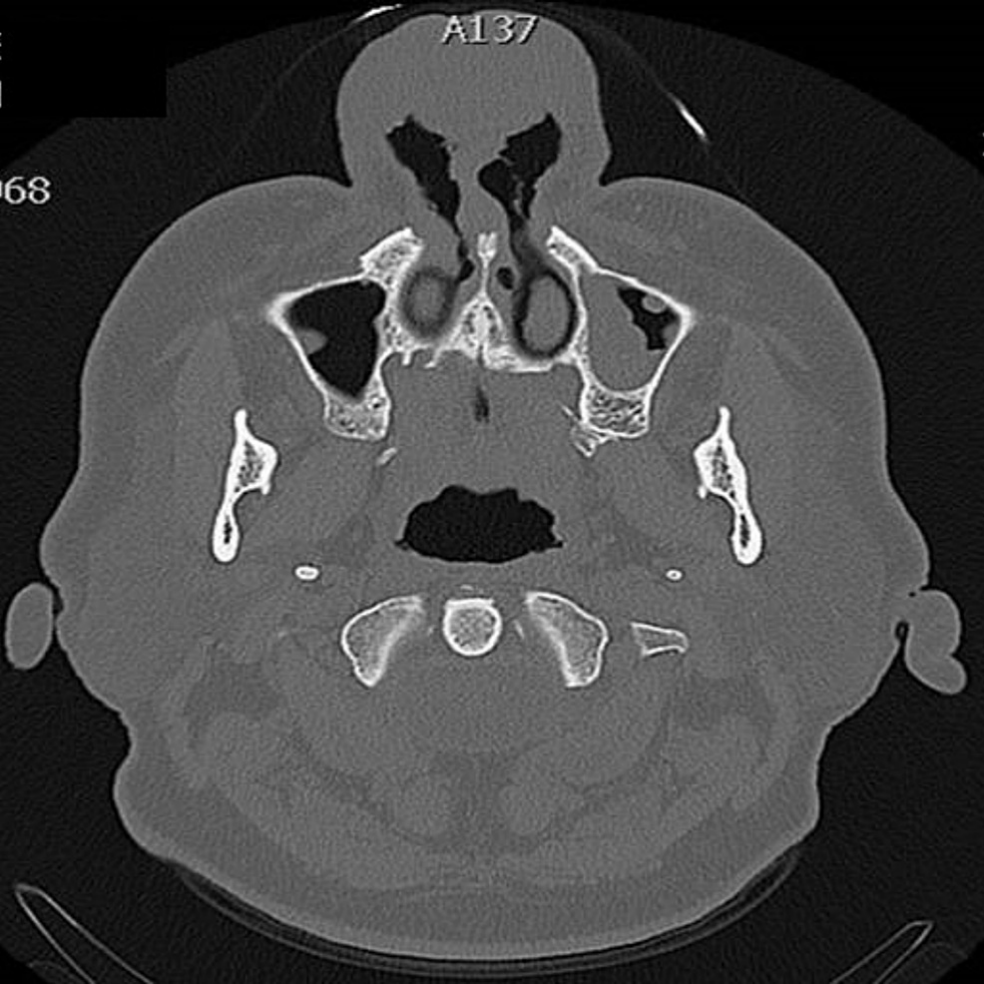
Axial computer tomography (CT) shows thickening of the skin, the subcutaneous fat and the cartilages of the nose.

In the computed tomography (CT) a significant thickening of the skin, the subcutaneous fat and the cartilages of the nose, was depicted (Figure 2). Thoracic and abdominal CT showed no lymphadenopathy or other pathological findings.

**Figure 3:**
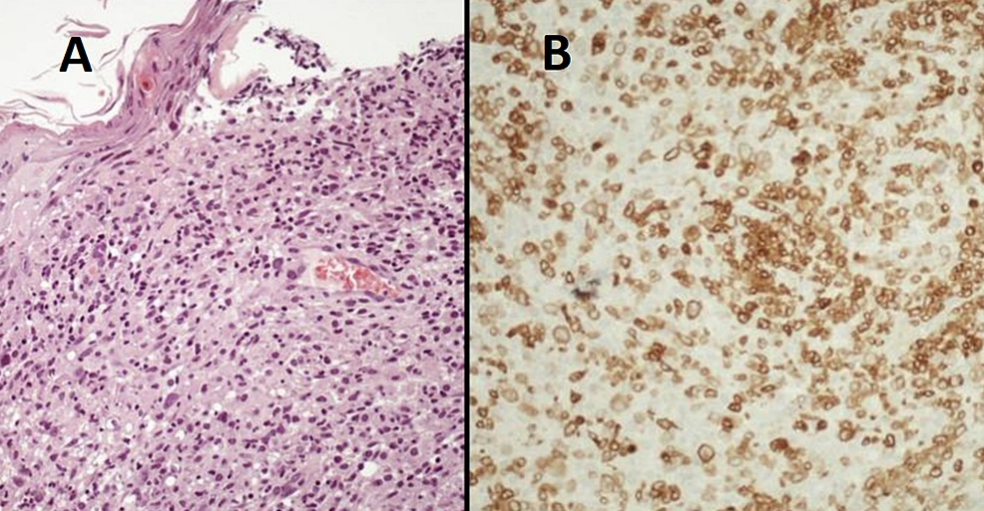
Tumor cells had a medium-large size, lymphoid nuclei with dispersed chromatin, and wide cytoplasms which exhibited a mature T-cell immunophenotype, most of them stained with CD30. Hematoxilin and eosin stain (A). An immunohistochemical study (B).

The patient underwent an excisional biopsy of the cutaneous lesion under local anesthesia with a diameter of 1 cm. The biopsy revealed an extensive area of necrosis, infiltration of atypical lymphoid cells of medium and large sizes in the dermis and in the subcutaneous tissue with considerable eosinophilia (Figure 3A, 3B). The special staining for Mycobacterium tuberculosis was negative. Immunohistochemically, tumor cells were positive for CD2, CD3, CD4, CD30, CD43 and they indicated negative staining for anaplastic lymphoma kinase (ALK), CD15, p63, CD1a, CD56, CD79a, CD5, CD7, CD20, PAX5, S-100, CK5/6. Subsequently, a bone marrow biopsy was conducted and the result did not demonstrate any pathology. The aforementioned test results were not compatible with the diagnosis of MF. Skin biopsy revealed primary cutaneous anaplastic-large cell lymphoma with CD30-positive staining (PC-ALCL).

**Figure 4:**
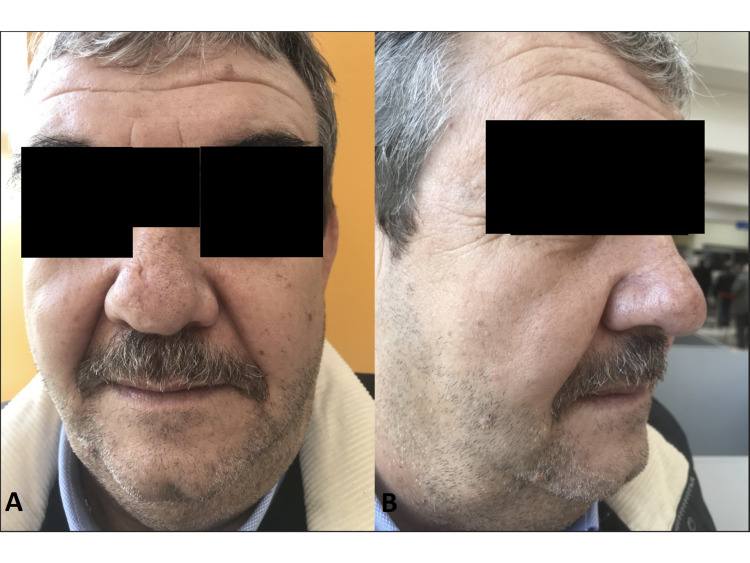
A/B: Complete spontaneous resolution observed after 6 months of follow-up.

The patient received intravenous ampicillin-sulbactam for 14 days as a primary treatment for the ulcer skin lesions according to the cultivations that were taken along with the skin biopsy. A corticosteroid ointment was also added for 20 days, for local treatment. As there is a lack of a multi-disciplinary team in many health care systems, similarly in our case, the pulmonologists insisted on the treatment with rifampin and ethabutol that was followed for six months based on the positive Mantoux test, even though the special staining for Mycobacterium tuberculosis was negative. Moreover, the hematologists did not suggest any kind of therapy (irradiation or chemotherapy). After two months, the lesion on the nasal skin and nasal cavity healed, leaving a minimal scar. Six months later, the patient remained free of local or systemic disease without any sign of recurrence (Figure 4A, 4B).

## Discussion

Primary cutaneous CD30+ lymphoproliferative disorders are the second most common group of cutaneous T-cell lymphomas. Given their rarity, these entities represent diagnostical and therapeutic challenges [7,9-10]. The PC-ALCL is typically seen in older males but it is infrequent in children and adolescents [8,11]. The clinical differential diagnosis, considered in our case, includes the infectious process, nasal tuberculosis, or a rapidly growing neoplasm such as the lethal midline granuloma (LMG) / nasal natural killer (NK) T-cell lymphoma (LMG-NTL). In our manuscript, this tumor was presented as a non-specific cutaneous lesion, so the excisional biopsy was the “gold standard” examination that could lead to the diagnosis. In addition, the nasal dorsum and tip location of the lesion required a differential diagnosis with a nasal-type killer (NK)/T-cell lymphoma. Both tumors are presented on the nasal skin with ulcerated papules and tissue necrosis. However, the NK/T-cell lymphoma results in thrombosis and epithelial hyperplasia. Furthermore, the neoplastic cells in NK/T-cell lymphoma express CD56 and are frequently associated with EBV infection. On the contrary, PC-ALCL is a disorder that expresses the CD30 antigens and it is characterized by the absence of EBV infection. The dual expression of CD30 and CD56 is unusual and is limited to ALCL. Regarding the prognosis, the NK/T-cell lymphoma is an aggressive tumor, whilst the PC-ALCL has a brilliant prognosis as in several cases it might be self-healing [12-13].

A diagnosis of PC-ALCL should be made only after the exclusion of systemic localizations by bone marrow examination and adequate staging procedures. It is frequently presented as one to multiple lesions greater than 2 cm in diameter, most often localized. Patients with PC-ALCL have a generally excellent prognosis, with a reported 10-year survival rate nearly exceeding 90% [5]. After the diagnosis, a follow-up for a period of four to six weeks is recommended, as 25% of lesions will regress. In the case that the disease would not regress spontaneously, for a solitary lesion, local radiotherapy with a dose of 40 Gy, would be the preferred treatment. Surgical excision is an alternative approach to localized lesions and can be associated with radiotherapy [7,14]. However, it has been reported that chemotherapy could not prevent future relapses of this lymphoproliferative disorder and dodging extracutaneous involvement [15]. Furthermore, it has been delineated that physical activity and improvement of the patients’ general condition would have a positive effect on the prognosis of the disease [16]. In our case, there was no need for radiotherapy or surgical treatment, as five weeks after the conservative therapy the skin lesions started regressing and nearly three weeks later they were healed [1]. The follow-up was performed every month with the last one at six months by otorhinolaryngologists, hematologists, and pulmonologists whereas a strong recommendation for a new control every year, was also given.

## Conclusions

In conclusion, PC-ALCL is a rare neoplasm developed de novo on the skin. It belongs to CD30+ lymphoproliferative disorders and the differential diagnosis is crucial because the spectrum of clinical and histological findings frequently overlaps with other conditions. Surgical excision and radiotherapy are the most common therapeutic programs for primary cutaneous ALCL while chemotherapy is used as therapy for multifocal cases. Due to the high risk of a future relapse the patients with PC-ALCL should be maintained in close observation and frequent assessment.

## References

[1] Kadin ME, Carpenter C (2003). Systemic and primary cutaneous anaplastic large cell lymphomas. Semin Hematol.

[2] Agnarsson BA, Kadin ME (1988). Ki-1 positive large cell lymphoma. A morphologic and immunologic study of 19 cases. Am J Surg Pathol.

[3] Benner MF, Willemze R (2008). Bone marrow examination has limited value in the staging of patients with an anaplastic large cell lymphoma first presenting in the skin. Retrospective analysis of 107 patients. Br J Dermatol.

[4] Willemze R, Jaffe ES, Burg G (2005). WHO-EORTC classification for cutaneous lymphomas. Blood.

[5] Liu HL, Hoppe RT, Kohler S, Harvell JD, Reddy S, Kim YH (2003). CD30+ cutaneous lymphoproliferative disorders: the Stanford experience in lymphomatoid papulosis and primary cutaneous anaplastic large cell lymphoma. J Am Acad Dermatol.

[6] Tomaszewski MM, Lupton GP, Krishnan J, May DL (1995). A comparison of clinical, morphological and immunohistochemical features of lymphomatoid papulosis and primary cutaneous CD30(Ki-1)-positive anaplastic large cell lymphoma. J Cutan Pathol.

[7] Diamantidis MD, Myrou AD (2011). Perils and pitfalls regarding differential diagnosis and treatment of primary cutaneous anaplastic large-cell lymphoma. ScientificWorldJournal.

[8] Marçal N, Campelos S, Dias L, Gonçalves M, Pereira G, Godinho T (2012). Primary cutaneous CD30-positive anaplastic large-cell lymphoma of the external auditory canal. Ear Nose Throat J.

[9] Martín JM, Ricart JM, Monteagudo C (2007). Primary cutaneous CD30+ anaplastic large-cell lymphomas mimicking keratoacanthomas. Clin Exp Dermatol.

[10] Salama S (2005). Primary "cutaneous" T-cell anaplastic large cell lymphoma, CD30+, neutrophil-rich variant with subcutaneous panniculitic lesions, in a post-renal transplant patient: report of unusual case and literature review. Am J Dermatopathol.

[11] Beljaards RC, Kaudewitz P, Berti E (1993). Primary cutaneous CD30-positive large cell lymphoma: definition of a new type of cutaneous lymphoma with a favorable prognosis. A European Multicenter Study of 47 patients. Cancer.

[12] Santiago-et-Sánchez-Mateos D, Hernández-Martín A, Colmenero I, Mediero IG, León A, Torrelo A (2011). Primary cutaneous anaplastic large cell lymphoma of the nasal tip in a child. Pediatr Dermatol.

[13] Mraz-Gernhard S, Natkunam Y, Hoppe RT, LeBoit P, Kohler S, Kim YH (2001). Natural killer/natural killer-like T-cell lymphoma, CD56+, presenting in the skin: an increasingly recognized entity with an aggressive course. J Clin Oncol.

[14] Savage KJ, Harris NL, Vose JM (2008). ALK- anaplastic large-cell lymphoma is clinically and immunophenotypically different from both ALK+ ALCL and peripheral T-cell lymphoma, not otherwise specified: report from the International Peripheral T-Cell Lymphoma Project. Blood.

[15] Kumar S, Pittaluga S, Raffeld M, Guerrera M, Seibel NL, Jaffe ES (2005). Primary cutaneous CD30-positive anaplastic large cell lymphoma in childhood: report of 4 cases and review of the literature. Pediatr Dev Pathol.

[16] Sica A, Vitiello P, Ronchi A (2020). Primary cutaneous anaplastic large cell lymphoma (pcALCL) in the elderly and the importance of sport activity training. Int J Environ Res Public Health.

